# Cation Incorporation and Synergistic Effects on the Characteristics of Sulfur-Doped Manganese Ferrites S@Mn(Fe_2_O_4_) Nanoparticles for Boosted Sunlight-Driven Photocatalysis

**DOI:** 10.3390/molecules27227677

**Published:** 2022-11-08

**Authors:** Sohail Nadeem, Mehak Bukhari, Mohsin Javed, Shahid Iqbal, Mirza Nadeem Ahmad, Hamad Alrbyawi, Murefah Mana Al-Anazy, Eslam B. Elkaeed, H. H. Hegazy, Muhammad Abdul Qayyum, Rami Adel Pashameah, Eman Alzahrani, Abd-ElAziem Farouk

**Affiliations:** 1 Department of Chemistry, School of Science, University of Management and Technology, Lahore 54770, Pakistan; 2 Department of Chemistry, School of Natural Sciences (SNS), National University of Sciences and Technology (NUST), H-12, Islamabad 46000, Pakistan; 3 Department of Applied Chemistry, Government College University, Faisalabad 38030, Pakistan; 4 Pharmaceutics and Pharmaceutical Technology Department, College of Pharmacy, Taibah University, Medina 42353, Saudi Arabia; 5 Department of Chemistry, College of Science, Princess Nourah bint Abdulrahman University, P.O. Box 84428, Riyadh 11671, Saudi Arabia; 6 Department of Pharmaceutical Sciences, College of Pharmacy, AlMaarefa University, Riyadh 13713, Saudi Arabia; 7 Department of Physics, Faculty of Science, King Khalid University, Abha P.O. Box 9004, Saudi Arabia; 8 Research Center for Advanced Materials Science (RCAMS), King Khalid University, P.O. Box 9004, Abha 61413, Saudi Arabia; 9 Department of Chemistry, Division of Science & Technology, University of Education, Lahore 54770, Pakistan; 10 Department of Chemistry, Faculty of Applied Science, Umm Al-Qura University, Makkah 24230, Saudi Arabia; 11 Department of Chemistry, College of Science, Taif University, P.O. Box 11099, Taif 21944, Saudi Arabia; 12 Department of Biotechnology College of Science, Taif University, P.O. Box 11099, Taif 21944, Saudi Arabia

**Keywords:** photocatalysis, cation doping, manganese ferrite, synergistic effects, dye

## Abstract

In the present work, sulfur-doped manganese ferrites S@Mn(Fe_2_O_4_) nanoparticles were prepared by using the sol-gel and citrate method. The concentration of sulfur varied from 1 to 7% by adding Na_2_S. The samples were characterized by performing Fourier Transformed Infrared Spectroscopy (FTIR), Energy Dispersive X-ray (EDX), X-ray diffraction (XRD), Scanning Electron Microscopy (SEM) and Ultraviolet–Visible spectroscopy (UV–Visible). The synthesized sulfur-doped manganese ferrites were applied to evaluate the photocatalytic degradation of the dyes. Further, the degradation studies revealed that the nanoparticles successfully degraded the methylene blue dye by adding a 0.006 g dose under the sunlight. The sulfur-doped manganese ferrite nanoparticles containing 3% sulfur completely degraded the dye in 2 h and 15 min in aqueous medium. Thus, the ferrite nanoparticles were found to be promising photocatalyst materials and could be employed for the degradation of other dyes in the future.

## 1. Introduction

Environmental pollution caused by dye pollutants is a major issue that is being faced by the world nowadays. These are being released into mainstream water without any treatment, causing colored, toxic, and polluted water. Among these dyes, some are hard to degrade, owing to their structure and their synthetic origin [1]. Moreover, they may also produce complex toxins or carcinogens after undergoing various secondary reactions (hydrolysis, oxidation, etc.) in the environment [2]. The discharge of leftover dyes has serious consequences for the environment and human health. Rhodamine B (RhB), for example, is a well-known cationic xanthene dye that has been widely utilized in printing, textile, and photography productions [3]. Because of their distinctive structures and aspects such as physiochemical features, Mn(III/IV) oxides have acquired a lot of interest in recent years as a way to eliminate organic dye pollution [4]. Numerous investigations have shown that different Mn(III/IV) oxides may remove dye by catalytic degradation and adsorption [5]. Dye removal may be significantly impacted by the crystalline forms of manganese oxides and the pH of the solution [6].

Water contamination is a severe problem that has a direct impact on our lives and is projected to worsen in the next decades. Many organic and inorganic contaminants, as well as microbial species, have been found in water [7]. Mineral extraction from water is having huge social, biological, economic, political, and environmental consequences [8,9,10]. In addition, owing to misuse in irrigation, the available groundwater level is rapidly diminishing [11].

Based on their place of origin, dyes may be divided into two categories: natural dyes and synthetic dyes [12]. Natural colors have been utilized in the past for quite some time, mainly in the textile-dying field. The expanding demand and exorbitant expenses of natural dye extraction induced the revelation of synthetic dye from petrochemical compounds [13,14]. Synthetic dyes are often employed in oil-based applications, as well as in paper printing, fabric coloring, and photography [15,16,17]. At present, they have overwhelming demand in the material market, with almost 8 × 10^5^ tons of production each year. This is because of their wide scope of pigment colors and steady coloration [18]. The textile industry releases an immense amount of water in dying processes, making it difficult to treat the massive amount of this hazardous wastewater [19].

Dyes released by textile enterprises represent a danger to people in general and ecological security [20]. Nowadays, the removal of dyes from effluents is refined by physio-chemical cycles [21]. Such techniques are definitely exorbitant, and dyes are taken out in the form of concentrated squanders that have disposal issues [22]. The degradation of textile colors with typical methods has acquired vitality, as these are less expensive and less useful as biodegradable technology [23].

For removing dyes from water, different approaches have been established to treat and recycle water contents [22]. The most significant of these approaches are reverse osmosis, ion exchange, electrodialysis, electrolysis, and adsorption [24]. Adsorption is a quick, economical, and general technique [25]. The advancement of economical adsorbents has prompted the fast development of research interests in this field. The traditional techniques include chemical treatment, filtration, absorption, precipitation, UV mechanism [26], and distillation. The mostly physical separation techniques are mechanical screening, hydrodynamics, concentration, flotation, soil-water density, and the hydrophilic characteristics of metals [27].

There are advanced oxidation processes (AOP) that degrade harmful contaminants in water through strong oxidants like hydroxyl radicals [28]. Magnetic catalytic application is one of the emerging fields in AOP to degrade these pollutants. This property of magnetic catalysts has allowed us to make use of them in engineering separation applications [29]. Separation can be done related to their nanostructures, as the properties would differ based on the atoms’ order in the magnetic structure [30]. Iron nanoparticles show strong ferromagnetic applications that are being broadly studied, and research is being done extensively in this field [31]. They are very effective and efficient in eradicating many contaminants in the environment, including both those that are organic and inorganic in nature. Low-intensity magnetic field application will stimulate the material’s magnetization and hence make the utilization of magnetic force possible. However, if there is an interruption in the magnetic field, then the concerned magnetization will be lowered to zero intensity. This, it is essential to consider the release of particles after waste adsorption [32].

The enhanced photocatalytic activities of many ferrites and metal doped ferrites have already been reported, such as nickel ferrites [33], titania-doped CoFe_2_O_4_ [34], manganese-doped cobalt ferrites [35] and many others [32,36,37,38]. In the current study, sulfur doped manganese ferrite nanoparticles were synthesized (3%, 5% and 7% S@Mn(Fe_2_O_4_)) via the sol-gel method for the degradation of methylene blue dye, which is the common dye contaminant present in the wastewater and responsible for many health hazards. The electron-rich S^-^ ions present in sulfur-doped manganese metal ferrites are involved in the electron transport between the dye and ferrites, which showed the resulting stable intermediate product and causing the metal NPs to remove the color of methylene blue. This approach has a commercial-scale application due to its non-hazardous nature. The main motive of this research was to combine the photocatalysts to reduce the recombination of pairs of e/h^+^. Taking two photocatalysts and combining them would effectively separate the e/h^+^ pairs, hence permitting more accessible species for the reduction and oxidation reaction with different contaminants of wastewater. Successful combinations of sulfur and manganese ferrite photocatalyst were analyzed by various analytical techniques, i.e., SEM, XRD FTIR, EDX, and UV–visible spectroscopy. Further, adsorption capacity and photocatalytic activity was also evaluated for the photocatalytic degradation of methylene blue dye. The significance and potential of novel ferrite nanoparticles were excellent in dye degradation and their application in photocatalysis.

## 2. Materials and Methods

### 2.1. Synthesis of Sulfur-Doped Manganese Metal Ferrites Nanoparticles

**Figure 1:**
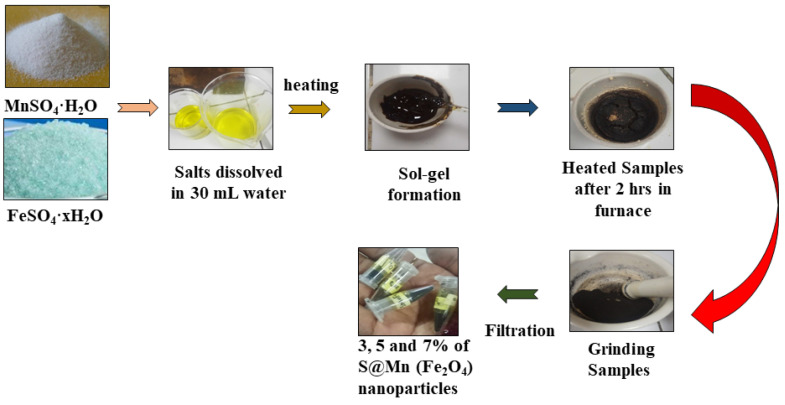
Synthesis of sulfur-doped manganese metal ferrites nanoparticles.

Sol-gel and the citrate method were used to synthesize the sulfur-doped manganese metal ferrites nanoparticles [39]. By mixing metal, iron precursors, and citric acid, the gel was produced [40]. To create a gel-like substance, iron precursors were dissolved in water and stirred mechanically [41]. Citric acid was added after the gel had formed, aiding in the equal dispersion of the metal ions throughout the solution (Figure 1). The combination gel was then dried and sintered in a heating furnace at various temperatures between 450 and 800 °C [42]. The sintering time was different, and then they were further examined to check the effects of their photocatalytic activity [43]. Sodium sulfide (Na_2_S), Fe(NO_2_)_3_·9(H_2_O), and Mn(NO_3_)_2_·4(H_2_O) were mixed completely and dissolved in 30 mL of distilled water, separately. This solution was then stirred for 20 min [44]. Then, the solution of citric acid was added to the above solution at 60 °C with magnetic stirring. After that, a polymerization agent, ethylene glycol, in 5 mL quantity was added dropwise to the solution [45]. The product, in solution form, was then dried at 120 °C to obtain a gel. Finally, the gels were dehydrated and then ground to obtain a fine powder of ferrite [46].

### 2.2. Preparation of Methylene Blue and Chromium (IV) Solution

For photocatalytic activity, a methylene blue solution was prepared by dissolving 0.006 g in 500 mL of distilled water. The prepared solution was sonicated for about 5 min and then placed in the dark for settling.

### 2.3. Characterization Methods

Various analytical techniques were used to characterize the samples and their catalytic potential. The dye solutions’ absorbance was measured using UV–visible spectroscopy (SHIMADZU UV-1700) [12]. To examine the structural features of the produced nanoparticles, Fourier-transform infrared spectroscopy (ALPHA BRUKER FTIR) was utilized. Particle morphology, size, and form were examined using scanning electron microscopy (FE1 Nova Nano SEM 450) [5]. The crystalline makeup and crystallite size were both evaluated with the help of X-ray diffraction (BRUKER D8 DISCOVER) and energy-dispersive X-ray (Oxford Inca XACT EDX) to study the characteristics of samples.

## 3. Results and Discussion

### 3.1. Scanning Electron Microscopy

**Figure 2:**
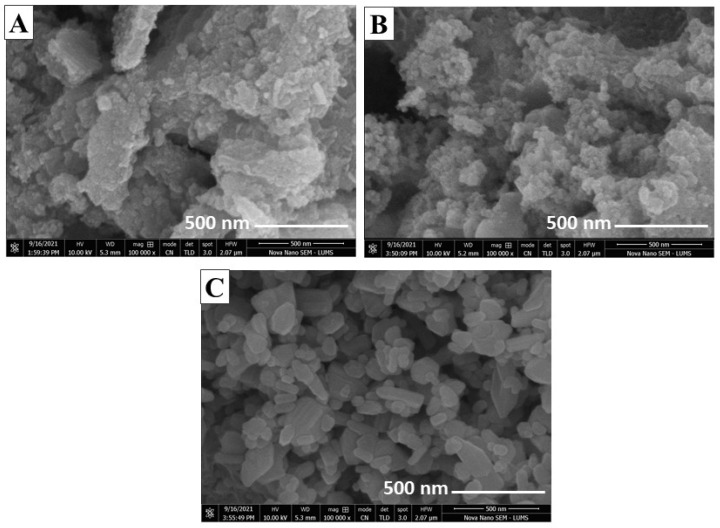
Surface morphology of S@Mn(Fe_2_O_4_) composites: (**A**) SEM of 3%S@Mn(Fe_2_O_4_), (**B**) SEM of 5%S@Mn(Fe_2_O_4_), and (**C**) SEM of 7%S@Mn(Fe_2_O_4_).

SEM images revealed the surface morphology of the prepared metal sulfide nanoparticles at a scale of 500 nm. Figure 2 shows SEM images of 3%, 5%, and 7%S@Mn(Fe_2_O_4_) composite nanoparticles. All images depicted the granular and crystalline structure of composites. The material’s range of particle size, according to SEM data, was between 50 and 100 nm.

### 3.2. Energy-Dispersive X-Ray Analysis (EDX)

**Figure 3:**
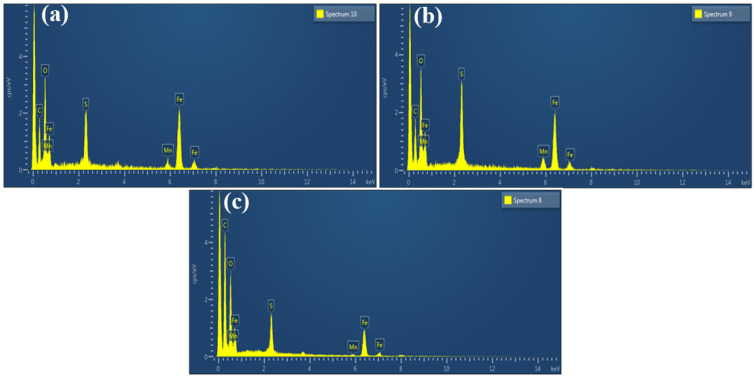
EDX spectrum of (**a**) 3%S@Mn(Fe_2_O_4_), (**b**) 0.05%S@Mn(Fe_2_O_4_), and (**c**) 0.07%S@Mn(Fe_2_O_4_).

EDX provided a good source of information about the newly synthesized material formulation and de-formulation. This technique was done to clarify an idea about the elements that would be present in the material, which is also called elemental analysis. Figure 3a shows the EDX result of the synthesized nanomaterial i.e., 3%S@Mn(Fe_2_O_4_). In Table 1, information about the elements present is given. In Figure 3a, peaks are shown due to the presence of elements, along with other peaks for other elements present.

In Table 2, information (apparent concentration and weight percent) about the present elements is reported. In Figure 3b, peaks of constituent elements are shown. It was also clear from the peak that sulfur was present in a higher amount in this material, in addition to other peaks present due to some elemental impurities. Figure 3c shows that peaks of different elements were present in the synthesized NPs. EDX revealed the peak of S, confirming that the substance contained sulfur [47]. The elemental peaks in Figure 3c indicate the presence of sulfur along with other elements, which are given in Table 3.

### 3.3. Fourier-Transform Infrared Spectroscopy

**Figure 4:**
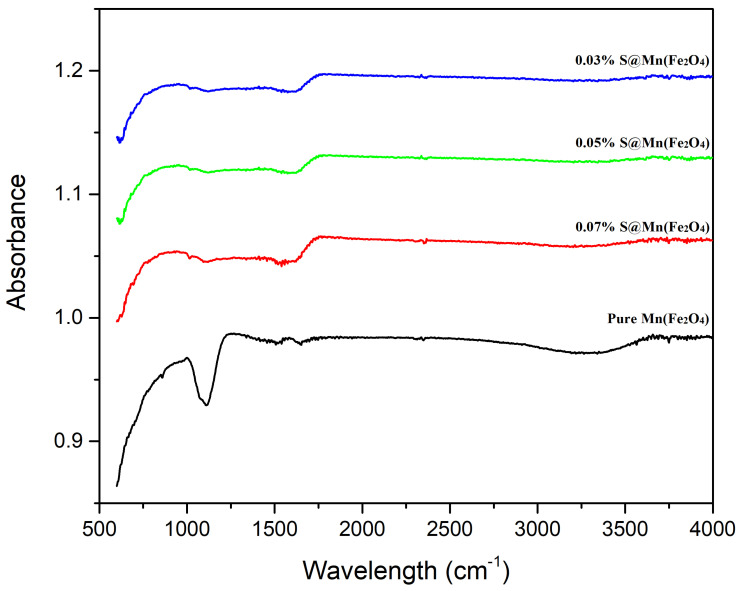
FTIR spectrum of S@Mn(Fe_2_O_4_) composites.

The prepared sulfur-doped manganese ferrites S@Mn(Fe_2_O_4_) nanoparticles were characterized by FTIR. Figure 4 shows the different peaks of S@Mn(Fe_2_O_4_) nanoparticles in the FTIR of the material. Due to the stretching vibration of the H-bonded O-H groups, a broad absorption band at 3400 cm^−1^ and a less intense band at 1620 cm^−1^ were observed. The metal-O band was present, which accounted for the peak at 600–500 cm^−1^ [48]. These peaks represented the various stretching frequencies of the many bonds forming the material’s atoms. The development of bonds in S@Mn(Fe_2_O_4_) NPs was seen in Figure 4. MnFe_2_O_4_ NPs’ stretching vibration appeared as the peak at 600 cm^−1^, and the S-O bond’s mild stretching vibration was attributed to the peak at 1047 cm^−1^. Mn(Fe_2_O_4_) NPs’ stretching vibration was shown by the peak at 615 cm^−1^ [49].

### 3.4. XRD

**Figure 5:**
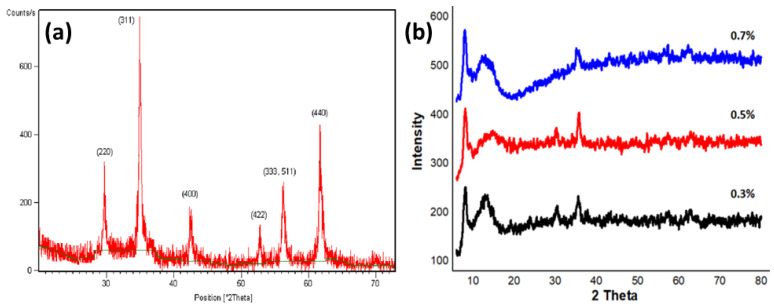
XRD pattern of (**a**) pure manganese ferrite powder and (**b**) S@Mn(Fe_2_O_4_) composite (0.3, 0.5, 0.7%).

By using XRD, the crystalline makeup of generated nanocomposites was investigated. In the XRD study, many diffraction peaks were seen. The spectrum’s strong and narrow peaks demonstrated the nanocomposite’s crystalline composition. Figure 5 showed the XRD result of sulfur-doped manganese ferrites nanoparticles, which described that these NPs were of hexagonal wurtzite crystal structure with high crystallinity. The findings showed that the spinel formation had the highest peak at 311 and the manganese ferrite peak had already been identified by Miller indices at (220), (222), (400), (422), (511), and (440).

### 3.5. UV–Vis Characterization of S@Mn(Fe_2_O_4_) Composite

The absorption properties of MnFe_2_O_4_ samples, 1% S@MnFe_2_O_4_, and 3% S@MnFe_2_O_4_ NPs’, were evaluated using the UV–vis spectrophotometer. When comparing MnFe_2_O_4_ and 1% S@ MnFe_2_O_4_ to 3 percent S@ MnFe_2_O_4_ NPs, light harvesting was increased from 290 nm to 740 nm. The 3% S@ MnFe_2_O_4_ NPs material’s enhanced photocatalytic efficiency was due to the incorporation of S with MnFe_2_O_4_. Additionally, a key factor in the efficiency of photocatalysis was the considerable improvement in light-harvesting effectiveness in the range of 440 to 740 nm [50,51].

**Figure 6:**
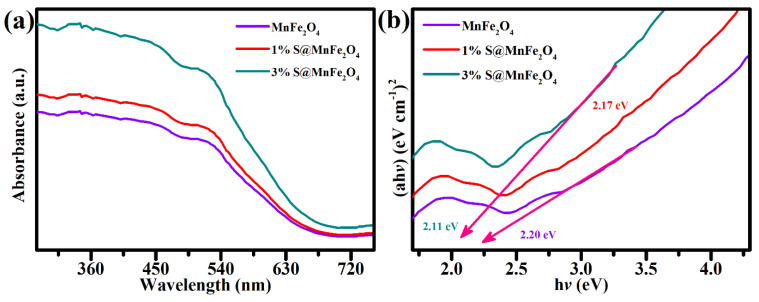
(**a**) UV–vis absorption measurements and (**b**) Tauc’s plots of MnFe_2_O_4_, 1% S@MnFe_2_O_4_, and 3% S@MnFe_2_O_4_.

The energy bandgap values of these synthetic photocatalysts was also determine by plotting UV–vis light-harvesting spectra using Tauc’s plot (Figure 6b). The predicted bandgap values for MnFe_2_O_4_, 1 percent S@MnFe_2_O_4_, and 3 percent S@MnFe_2_O_4_ NPs were 2.20 eV, 2.17 eV, and 2.11 eV, respectively, as shown in Figure 6b. When compared to MnFe_2_O_4_ and 1% S@MnFe_2_O_4_, the energy bandgap of NPs made up of three percent S@MnFe_2_O_4_ decreased from 2.20 eV to 2.11 eV. The drop in bandgap values might be attributed to the effective surface combination of both components, which significantly increased the capabilities of the binary photocatalytic system. The lowered optical bandgap edge of 3-percent S@MnFe_2_O_4_ under visible light brightness may be related to the photocatalytic capabilities of MnFe_2_O_4_ and 1-percent S@MnFe_2_O_4_.

### 3.6. Photocatalytic Performance

**Figure 7:**
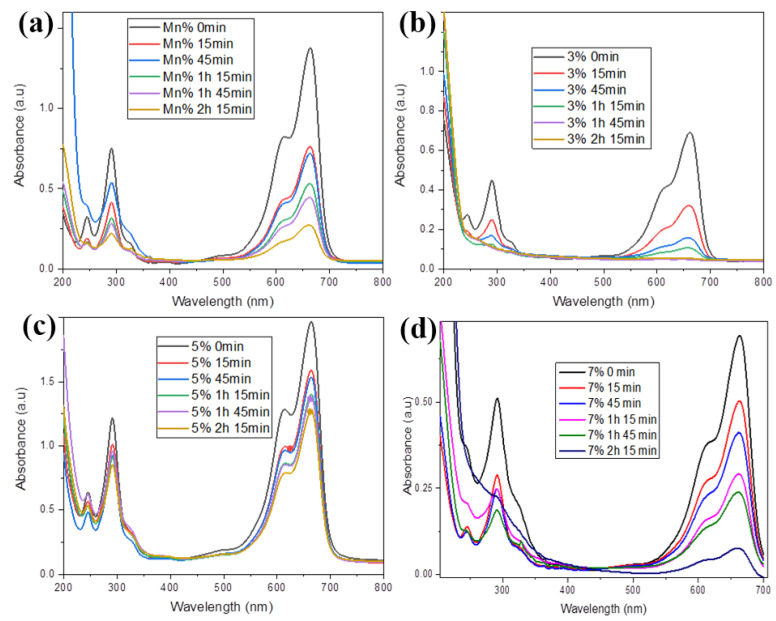
Photocatalytic assessment at different time intervals of (**a**) MnFe_2_O_4_, (**b**) 3%S@Mn(Fe_2_O_4_), (**c**) 5%S@Mn(Fe_2_O_4_), and (**d**) 7%S@Mn(Fe_2_O_4_).

To study the photodegradation of standard methylene blue, UV–visible spectroscopy was employed to evaluate the photocatalytic activity of the produced nanoparticles. After 10 min, UV absorption spectra (200–800 nm) were recorded for all the synthesized samples. The prepared sample was placed in sunlight, and afterward, the result of each percentage was compared for dye degradation to select the best catalyst. The photocatalytic activity of pure manganese ferrites (Figure 7a) and sulfur-doped manganese ferrites (Figure 7b–d) at different time intervals was executed separately. Thus, with the passage of time and with greater light intensity, dye degradation concentration was increased by the decrease in absorbance

Then, 0.006 g of the MB dye was dissolved in 500 mL of distilled water in a flask, from which 100 mL of solution was taken separately. It was then placed in the dark for about 30 min to maintain its adsorption–desorption equilibrium. After this, the degradation was carried out by placing the Petri dish in sunlight, and 5 mL of this dye solution was taken in a sample vial after regular time intervals. Similarly, four samples were collected, and it was found that there was no appreciable change in the UV–visible spectrum of MB without a catalyst.

The photocatalytic activity of manganese ferrites at different time intervals was executed separately. A comparison of results at different time intervals is shown in Figure 7a that depicted that the absorption level at the start was much greater than the degradation. Additionally, the photocatalytic activity of manganese ferrites with three percent sulfur doping was carried out individually at various time intervals. A comparison of results at different time intervals is shown in Figure 7b, which shows that the absorption level at the start was much greater than the degradation. When exposed to sunlight, the 3% of sulfur-doped manganese ferrite in 2 h and 15 min duration mitigated the MB to 100% in the sample solutions. These results implied that the addition of S to the Mn(Fe_2_O_4_) NPs significantly improved the dye degradation process. S@Mn(Fe_2_O_4_) NPs with a three percent composition showed high photocatalytic activity. Separate tests were performed on the photocatalytic activity of five percent sulfur-doped manganese ferrites at various time intervals (Figure 7c). A comparison of results at different time intervals is shown in Figure 7d, which shows that the absorption level at the start was much greater than the degradation. Separate tests were done on the photocatalytic activity of manganese ferrites containing 7% sulfur. A comparison of results at different time intervals is shown in Figure 7d, which reveals that the absorption level at the start was much greater than the degradation. All samples showed good adsorption at 200–250 nm, but their capability of absorption was increased and decreased depending on the time and doping of sulfur.

**Figure 8:**
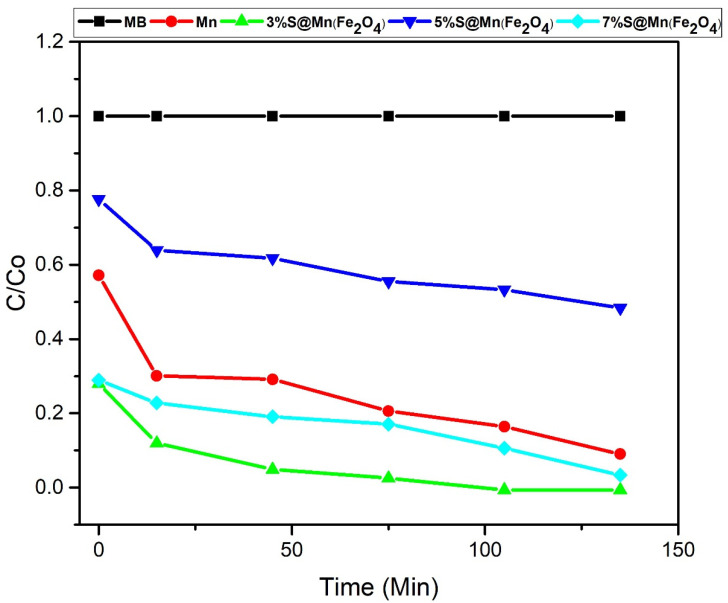
Photocatalytic degradation rate (C_e_/C_o_ vs. different time intervals).

The removal rate of pure manganese ferrite (NiS, SGCN) and three different concentrations of sulfur-doped manganese ferrite were compared by plotting Ce/Co versus time of irradiation (min). Results clearly showed that the highest degradation rate was exhibited by three percent S@Mn(Fe_2_O_4_) as compared to other samples. The graphs obtained are shown in Figure 8.

**Figure 9:**
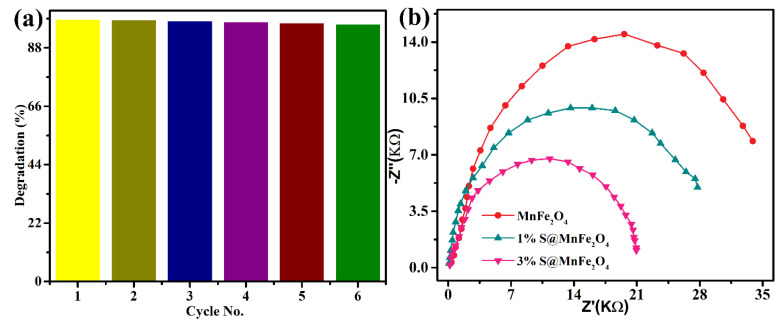
(**a**) the 3%S@Mn(Fe_2_O_4_) photocatalysts’ cyclic stability up to the 6th cycle and (**b**) EIS Nyquist plots of Mn(Fe_2_O_4_), 1%S@Mn(Fe_2_O_4_), and 3%S@Mn(Fe_2_O_4_).

**Figure 10:**
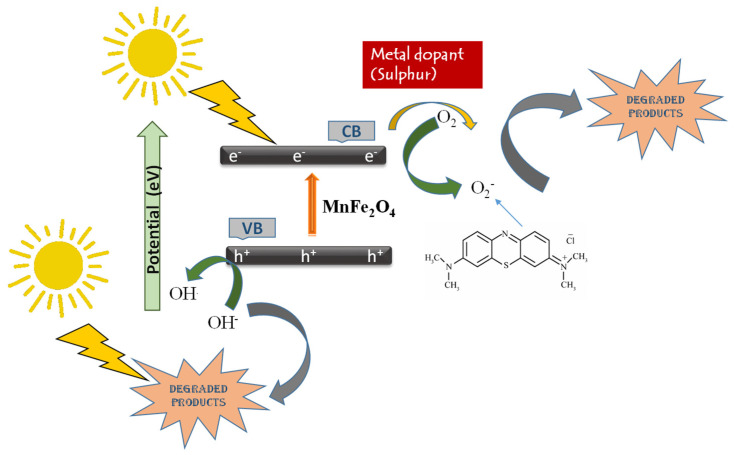
Mechanism of the photocatalytic activity of manganese ferrite in the presence of sunlight.

The three percent S@Mn(Fe_2_O_4_) catalysts’ stability was tested by gradually reducing MB up to six times. The results showed effective dye degradation, which was maintained at over 94 percent even after the sixth cycle (Figure 9a). However, after the first cycle, the catalytic rates were dramatically decreased. A possible explanation for the small decrease in catalytic effectiveness included the partial blockage of active sites. For this reason, the three percent S@Mn(Fe_2_O_4_) catalyst might function as a reliable, effective, and reusable photocatalytic system. The heterointerface charge transfer rate was evaluated using EIS in the dark at the electrode–electrolyte junction. A faster interfacial photoinduced charge transfer and departure efficiency were often associated with a narrower arc radius and lower electron transport resistance. The three percent S@Mn(Fe_2_O_4_) sample had the lowest charge-transmission resistance of all of the synthesized samples (Figure 9b). It illustrated the heterointerface contact of the three percent S@Mn(Fe_2_O_4_), which significantly supported electron transmission, increasing electron consumption and improving photocatalytic efficiency. Measurements of transient photocurrent were consistent with the EIS findings. The aforementioned experimental findings indicate that a three percent S@Mn(Fe_2_O_4_) heterojunction might greatly increase heterointerface electron transmission, the effective separation of photogenerated e- and h+ couples, and light-harvesting capabilities. The constructed three percent S@Mn(Fe_2_O_4_) photocatalytic mechanism under visible light is depicted in Figure 10.

## 4. Conclusions

The synthesis of sulfur-doped manganese metal ferrites was successfully carried out by sol-gel and the citrate method. Three samples having various concentrations (3%, 5%, and 7%) of sulfur-doped manganese metal ferrites were prepared using salts of manganese II sulfate and iron (II) sulfate following gel formation. These samples were then characterized by SEM to check their morphology, which exhibited a granular and crystalline structure. FTIR revealed the structural features and characteristic bonds developed in NPs. EDX data established the formation of sulfur-doped manganese metal ferrites by confirming the presence of sulfur. It also indicated the successful combination of both components, which enhanced the ability of the binary photocatalytic system. XRD analysis revealed that sulfur-doped manganese metal ferrites had a crystalline structure. The optical energy bandgap for sulfur-doped manganese metal ferrites was dropped from 2.20 eV to 2.11 eV as investigated by the UV–vis spectrum. Moreover, the photocatalytic activity of ferrites NPs was evaluated for checking the methylene blue dye degradation. It was also supported by the experimental findings such as the adsorption capacity and the photocatalytic activity. Among all the percentages, three percent sulfur-doped manganese ferrite was most efficient at mitigating 100% of methylene blue with 2 h and 15 min of sunlight exposure. These results confirmed the significance and potential of novel ferrite nanoparticles toward dye degradation and application in photocatalysis.

## Figures and Tables

**Table 1:** EDX data of 3%S@MnFe_2_O_4_ nanoparticles.

Element	Apparent Concentration	Wt.%
C	5.05	24.84
O	21.69	22.7
S	5.57	6.95
Mn	1.94	2.88
Fe	29.25	42.62
Total:		100

**Table 2:** EDX data of 5%S@MnFe_2_O_4_ nanoparticles.

Element	Apparent Concentration	Wt.%
C	4.42	23.94
O	22.23	24.01
S	7.17	9.08
Mn	4.51	6.82
Fe	24.25	36.14
Total:		100

**Table 3:** EDX data of 7%S@MnFe_2_O_4_ nanoparticles.

Element	Apparent Concentration	Wt.%
C	15.97	46.51
O	23.86	28.97
S	4.21	4.5
Mn	0.56	0.76
Fe	14.41	19.26
Total:		100

## Data Availability

The data will be available on request.
